# Toxoplasmosis in Cancer Patients and Suggestion for Screening

**DOI:** 10.31557/APJCP.2019.20.4.985

**Published:** 2019

**Authors:** Raafat Abdel Raafat Abdel, Rita Wassef, Enas Rizk, Hoda Sabry, Nevine Tadros, Abdallah Boghdady

**Affiliations:** 1 *1Department of Clinical Oncology, *; 3 *Department of Medical Parasitology, Kasr Al-Ainy Faculty of Medicine, Cairo University,*; 2 *Department of Medical Parasitology, Faculty of Medicine, Helwan University,*; 4 *Department of Medical Parasitology, Theodor Bilharz Research Institute, Egypt. *

**Dear Editor**

Dear Editor, we read the publication on “Toxoplasmosis an Overlooked Disease: Seroprevalence in Cancer Patients” with a great interest (Abdel Malek et al., 2018). Abdel Malek et al., (2018) “*Cancer patients showed a significantly higher rate of infection with T. gondii. For that reason, we recommend the inclusion of a screening test for toxoplasmosis in their routine workup.*” It is interesting that this study use a less controls comparing to the studied cancer patients in seroprevalent rate study. Whether there is any selection bias is an interesting question. In fact, toxoplasmosis is an important infection and the asymptomatic seropositive rate is not uncommon in developing countries such as India (Mohan et al., 2002). Whether the cancer contribute to increase risk of getting toxoplamosis is an interesting question. In many reports, the high prevalent rate of seropositivity against toxoplasmosis are reported. Meta-analysis also showed that the cancer patients, especially for those who have immunosuppression status usually have seropositivity to toxoplasmosis (Jiang et al., 2015). However, an interesting concern is there has never been identified specific pathophysiological process on increased severity of toxoplasmosis in cancerous patients comparing to that seen in infection in normal non-cancer subjects. In endemic areas, such as tropical developing countries, seroprevalence of toxoplasmosis is high and the asymptomatic infection is common (Gohardehi et al., 2018). Due to the high seropsiotive asymptomatic infection, the routine screening for toxoplasmosis has never been suggested in the endemic developing countries. It is questionable on the exact usefulness of implementation of toxoplasmosis screening among cancer patients. This issue is interesting and should be further systematically investigate on advance and cost - utility. 

## Conflict of interest

None.

## References

[B1] Abdel Malek R, Wassef R (2018). Toxoplasmosis an overlooked disease: seroprevalence in cancer patients. Asian Pac J Cancer Prev.

[B2] Gohardehi S, Sharif M, Sarvi S 2018) The potential risk of toxoplasmosis for traffic accidents: A systematic review and meta-analysis. Exp Parasitol.

[B3] Jiang C, Li Z, Chen P, Chen L (2015). The seroprevalence of Toxoplasma gondii in Chinese population with cancer: A systematic review and meta-analysis. Medicine (Baltimore).

[B4] Mohan B, Dubey ML, Malla N, Kumar R (2002). Seroepidemiological study of toxoplasmosis in different sections of population of Union Territory of Chandigarh. J Commun Dis.

