# Supplementary material for: Toxoplasmosis in Cancer Patients and Suggestion for Screening
Source: Asian Pac J Cancer Prev. 2019;20(4):985–6. doi: 10.31557/APJCP.2019.20.4.985 (PMC6948892; doi:10.31557/APJCP.2019.20.4.985)
Supplement: Related texts

# Reply: Dear Editor

**Published:** 2019

**Authors:** Beuy Joob, Viroj Wiwanitkit

**Affiliations:** 1 *Sanitation 1 Medical Academic Center, Bangkok, Thailand,*; 2 *Honorary professor, dr DY Patil University, Pune, India.*

**Dear Editor**

Toxoplasmosis can result in serious complications in the immunocompromised patients as AIDS, cancer, and transplant patients. This is due to reactivation of a latent infection or due to acquisition of a new infection (Ahmadpour, 2014).

Are immunocompromised patients as cancer patients more prone to acquire the infection than immunocompetent individuals? This question has not been answered yet. As a first step towards finding an answer to this question we conducted our study published in your prestigious journal (Abdelmalek et al., 2018) aiming to determine if there is a difference in the prevalence rate between cancer patients and non-cancerous patients. Our study proved that there is a significant statistical difference between patients and controls. Further studies are on the go.

We considered four groups of patients, each contained 50 patients. So, the control group is similar in number to each group of patients. Still, the total number of patients is bigger than that of the control which is not uncommon in prevalence studies (many are referred to in our paper). This is acceptable as long as statistical analysis of the results are done.

Regarding the addressed question of the necessity of screening of cancer patients based on our results in developing countries; we are confident that prevention of serious complications of toxoplasmosis by performing a simple screening test that costs approximately 1 USD is cost beneficial especially that we found a high prevalence of infection in our study and we aim to highlight this to all clinicians especially in developing countries. 

## References

[BA1] Ahmadpour E, Daryani A, Sharif M (2014). Toxoplasmosis in immunocompromised patients in Iran: a systematic review and meta-analysis. J Infect Dev Ctries.

[BA2] Abdel Malek R, Wassef R, Rizk E (2018). Toxoplasmosis an overlooked disease: Seroprevalence in cancer patients. Asian Pac J Cancer Prev.
